# Analyses of electronic health records utilization in a large community hospital

**DOI:** 10.1371/journal.pone.0233004

**Published:** 2020-07-01

**Authors:** Gautam Verma, Alexander Ivanov, Francis Benn, Anil Rathi, Nathaniel Tran, Ashwad Afzal, Parag Mehta, John F. Heitner

**Affiliations:** Department of Medicine, NewYork-Presbyterian Brooklyn Methodist Hospital, Brooklyn, New York, United States of America; University of California San Diego, UNITED STATES

## Abstract

**Introduction:**

The Electronic Health Record (EHR) has become an integral component of healthcare delivery. Survey based studies have estimated that physicians spend 4–6 hours of their workday devoted to EHR. Our study was designed to use computer software to objectively obtain time spent on EHR.

**Methods:**

We recorded EHR time for 248 physiciansover 2 time intervals. EHR active use was defined as more than 15 keystrokes, or 3 mouse clicks, or 1700 "mouse miles" per minute. We recorded total time and % of work hours spent on EHR, and differences in those based on seniority. Physicians reported duty hours using a standardized toolkit.

**Results:**

Physicians spent 3.8 (±2) hours on EHR daily, which accounted for 37% (±17%), 41% (±14%), and 45% (±12%) of their day for all clinicians, residents, and interns, respectively. With the progression of training, there was a reduction in EHR time (all p values <0.01). During the first academic quarter, clinicians spent 38% (± 8%) of time on chart review, 17% (± 7%) on orders, 28% (±11%) on documentation (i.e. writing notes) and 17% (±7%) on other activities (i.e. physician hand-off and medication reconciliation). This pattern remained unchanged during the fourth quarter.

**Conclusions:**

Physicians spend close to 40% of their work day on EHR, with interns spending the most time. There is a significant reduction in time spent on EHR with training and greater experience, although the overall amount of time spent on EHR remained high.

## Introduction

Electronic health record (EHR) has had a marked increase in penetrance in the United States health care since the passing of the Affordable Care Act (ACA) [1]. Numerous advantages offered by EHR (i.e., clear legibility, easy access to old records, and electronic prescribing) have made it an integral part of healthcare [2]. Adoption of different aspects of EHR, like computerized physician order entry and integration with imaging systems has led to an increasing reliance on the EHR for day to day clinical practice.

Although EHRpromisesa number of advantages, there are also many challenges which include a slow learning curve, high costs and difficulty with Health Information Exchangebetween systems [3]. Studies exploring EHR use emphasized extensive time as one of the significant drawbacks to EHR [4, 5]. It has been reported that physicians spend up to 6 hours a day on EHR in the hospital setting alone [4–7]. The added time required to navigate the EHR system has added to the long work hours for physicians and has been shown to negatively impact patient care, quality and efficiency through physician burnout [8–11]. A recent study has shown physician burnout to be both significantly higher than other professions and increasing over the past decade [12].

Despite the high prevalence of EHR use in the United States, there are few studies documenting the precise time physicians spend on EHR. Studies that have been performed have relied largely on self-reporting of work hours [13–15]. Our study sought to objectively record data to illustrate how much total time is spent using EHR. Additionally, we explored if this time differed by various levels of clinical experience and if any relationship exists between hours worked as it pertains to resident and patient satisfaction.

## Methods

Our study was conducted at NewYork-Presbyterian Brooklyn Methodist Hospital, a 651-bed tertiary care center. The study period extended from June 2016 to October 2016 and was approved by the institutional review board at New York Presbyterian- Brooklyn Methodist Hospital with waived consent process. We sampled 2 two week periods. One was early in the academic year (first academic quarter) and one, at the very end of it—fourth academic quarter. During these periods, the average hospital census remained above 95%. We used data from patients admitted to the medicine service including patients admitted to a units witha higher level of care unitWe thought that using “encounters” will be a better way to capture initial and subsequent daily patient-physician interactions and corresponding progress notes. We also performed a sub-study evaluating attending physicians with the following parameters: specialty, teaching position, number of patient encounters, and total time spent on EHR. Time per patient was calculated dividing total EHR time by the number of patient encounters. We used average time to better characterize different patient populations and have higher internal validity.

EHR time was determined by computerized software using CERNER^®^ (North Kansas City, MO). EHR active use was defined by more than 15 keystrokes, or 3 mouse clicks, or 1700 "mouse miles per minute”. The accepted definition of the mouse mile is a measure of distance between two points travelled by mouse, with 1 mouse mile = 1 pixel [7]. Inactive EHR use was defined as any track time outside of active EHR use and was excluded from our analysis. Active EHR usage time was recorded per user for each patient encounter. EHR usage included time spent on documentation, orders, reviewing charts, and miscellaneous tasks. During these same intervals, each resident and fellow self-reported the amount of hours spent at work via duty hour collection for the residency program. Hospitalist hours worked on a shift basis and hours were collected based on their shifts.

During the study, patients completed a five-question survey (S1 Table). Three of these questions were obtained from the Hospital Consumer Assessment of Healthcare Providers and Systems (HCAHPS) survey regarding “Your Care from Doctors.” These questions were answered on a numerical scale from 1–4, which ranged from answer choices of “never” to “always.” Two additional questionswere included in the study in an attempt to quantify direct patient-physician interaction: 1) “On average, how many minutes per day in total do you feel your resident doctor spends with you in person?” and 2) “On a scale of 1–100 (100 being the most satisfied), how satisfied are you with the amount of time spent with you by your resident doctor? (S1 Table). We recorded each patient’s estimation of how much face-to-face time was spent with their physician and how satisfied they were with this quantity. Any patient that was awake and alert and able to complete the survey was included in the study. Every resident physician who took direct care of a surveyed patient completed the Oxford Happiness Questionnaire (S2 Table).

### Statistical analysis

All statistical tests were two-tailed and p <0.05 was regarded as significant. STATA 14.2 (StataCorp, Texas, USA) was used to perform the statistical analysis. Continuous data were expressed as mean±standard deviation (SD), or in cases where the distribution is not normal, as median and the 25th and 75th percentiles. Comparisons of continuous data between groups were made using one-way or two-way analysis of variance or the Wilcoxon Rank Sum test as appropriate. The chi square test was used to make between group comparisons of discrete data. We used analysis of covariance for PGY level to access correlations between time on EHR with patient, or physician satisfaction. Number of hours a day was calculated accounting for days at work (12 days for residents, 10 for fellows and 7 for hospitalists).

## Results

During the study, a total of 248 clinicians were included in the analysis: In the first academic quarter there were 130 clinicians—102 residents (40 PGY-1, 29 PGY-2, and 33 PGY-3), 24 fellows, and 4 attending hospitalists. In the fourth academic quarter there were 118 clinicians—95 residents (34 PGY-1, 33 PGY-2 and 28 PGY-3), 19 fellows and 4 attending hospitalists. In the sub-study, we analyzed data for 188 attending from 12 different medicine sub-specialties.

**Fig 1:**
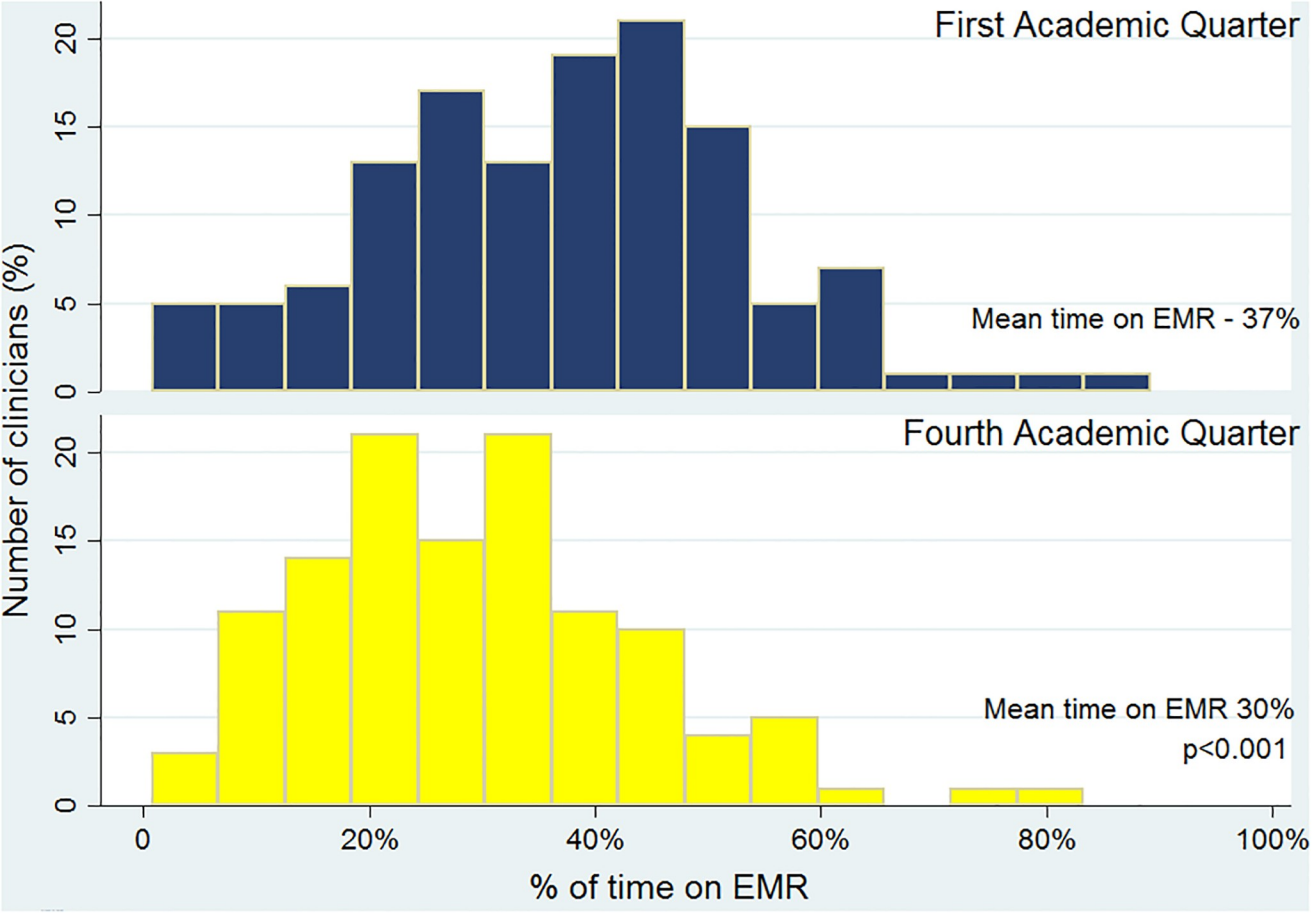
Percentage of time on EMR for all clinicians.

**Fig 2:**
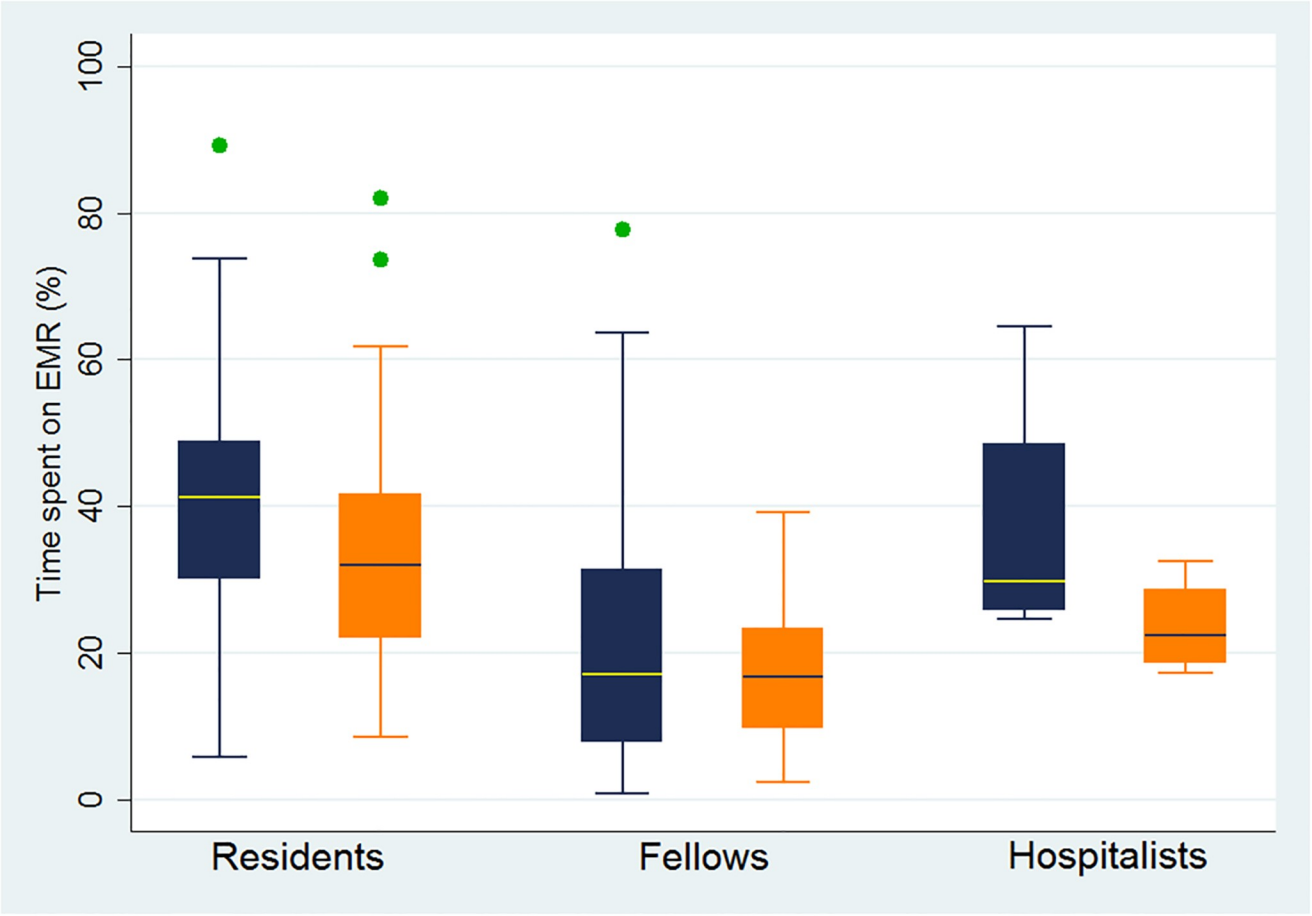
Time spent on EMR daily (%) and clinician training. Blue—First academic quarter; Orange—Fourth academic quarter.

In the first academic quarter, the average time spent by clinicians on EHR was 3.8 (±2) hours a day, or 37% (±17%), of their total work hours. When analyzing utilization of EHR based on the level of training; residents, fellows, and hospitalists spent 41% (±14%), 23% (±19%), and 37% (±19%) of their works hours on EHR, respectively. During the fourth academic quarter, clinicians were spending less time on EHR averaging 2.9 hours a day or 30% of their time (p<0.01) (Fig 1). This reduction was driven by residents (p<0.01) with no difference noted among fellow and attending physicians (Table 1, Fig 2).

**Table 1:** Time spent on EMR by clinician seniority.

	Residents	Fellows	Hospitalist	p r&f	p r&h	p f&h
	Hr daily/% on EMR	Hr daily/% on EMR	Hr daily/% on EMR			
First Academic Quarter	4.1 (±1.9)/41% (±14%)	2.0 (±1.7)/23% (±19%)	4.5(±2.2)/37% (±19%)	<0.01	0.67	0.08
Fourth Academic Quarter	3.1 (±1.5)/32% (±14%)	1.6 (±0.9)/17% (±10%)	2.8 (±0.8)/24% (±7%)	<0.01	0.21	0.40
P value for difference between Quarters	<0.01	0.67	0.15			

p r&f—comparing hours daily on EMR for resident and fellows

p r&h—comparing resident and hospitalists

p f&h—comparing fellow and hospitalists

**Fig 3:**
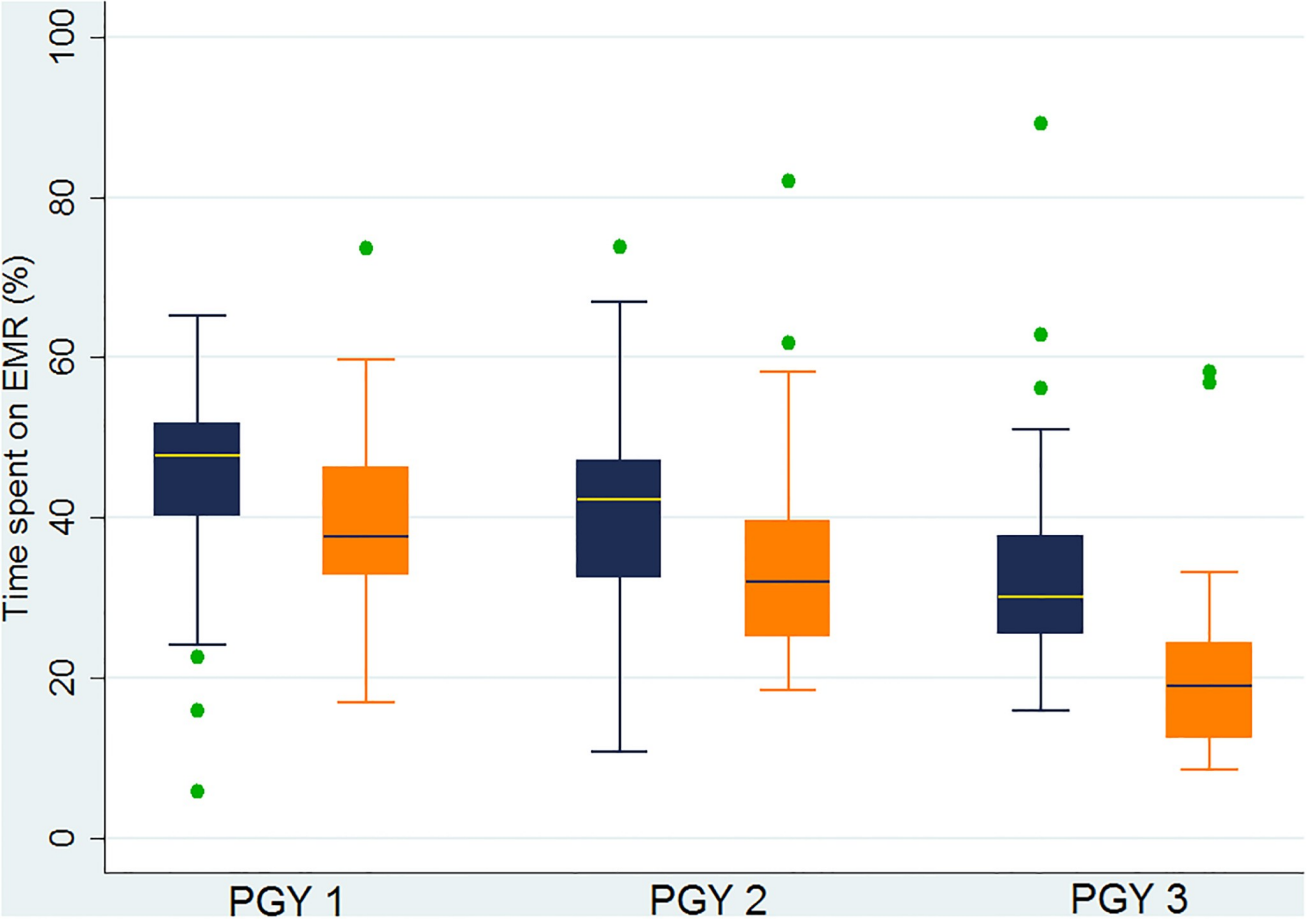
Time spent on EMR daily (%) and resident seniority. Blue—First academic quarter; Orange—Fourth academic quarter.

When analyzed by resident seniority, during the first academic quarter, PGY-1’s spent 45% (±12%) of their total work hours using EHR, while PGY-2’s and PGY-3’s spent 42% (±14%) and 34% (±16%), respectively. Comparing first and fourth academic quarters, it was noted that senior residents (PGY-2&3) were spending less time on EHR during the fourth academic quarter (p<0.04) with a trend toward less time on EHR among interns (p = 0.07). Throughout the year, PGY-1’s and PGY-2’s were spending more time on EHR than PGY-3’s (p values<0.04) (Table 2, Fig 3).

**Table 2:** Time spent on EMR based on post graduate year.

	PGY-1	PGY-2	PGY-3	p 1&2	p 1&3	p 2&3
	Hr daily/% on EMR	Hr daily/% on EMR	Hr daily/% on EMR			
First Academic Quarter	4.8 (±1.9)/45% (±12%)	4.6 (±2.0)/42% (±14%)	2.7 (±1.5)/34% (±16%)	0.33	<0.01	<0.04
Fourth Academic Quarter	4.0 (±1.5)/40% (±11%)	3.4 (±1.2)/34% (±13%)	1.8(±0.9)/21% (±12%)	0.08	<0.01	<0.01
P value for difference between Quarters	<0.01	0.04	<0.01			

p 1&2—comparing hours daily on EMR for PGY-1 and PGY-2.

p 1&3—comparing PGY-1 and PGY-3.

p 2&3—comparing PGY-2 and PGY-3.

**Fig 4:**
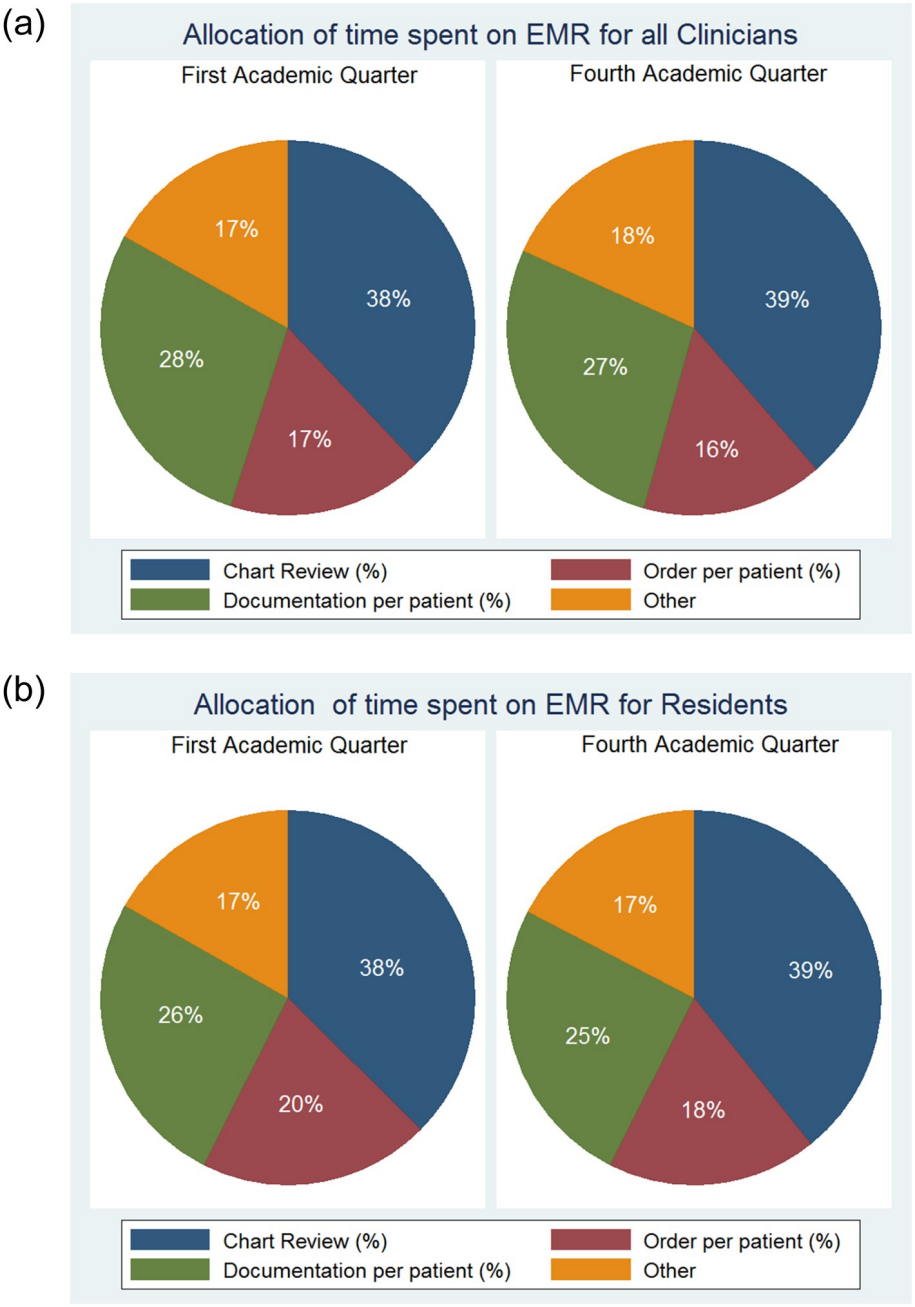
(a) Allocation of time on EMR for all Clinicians. (b) Allocation of time on EMR for all Residents.

During the first academic quarter, clinicians spent 38% (± 8%) of time on chart review, 17% (± 7%) on orders, 28% (±11%) on documentation (i.e. writing notes) and 17% (±7%)on other activities (i.e. physician hand-off and medication reconciliation). There are no significant differences in time allocation on EHR based on seniority or academic quarter (Fig 4).

**Fig 5:**
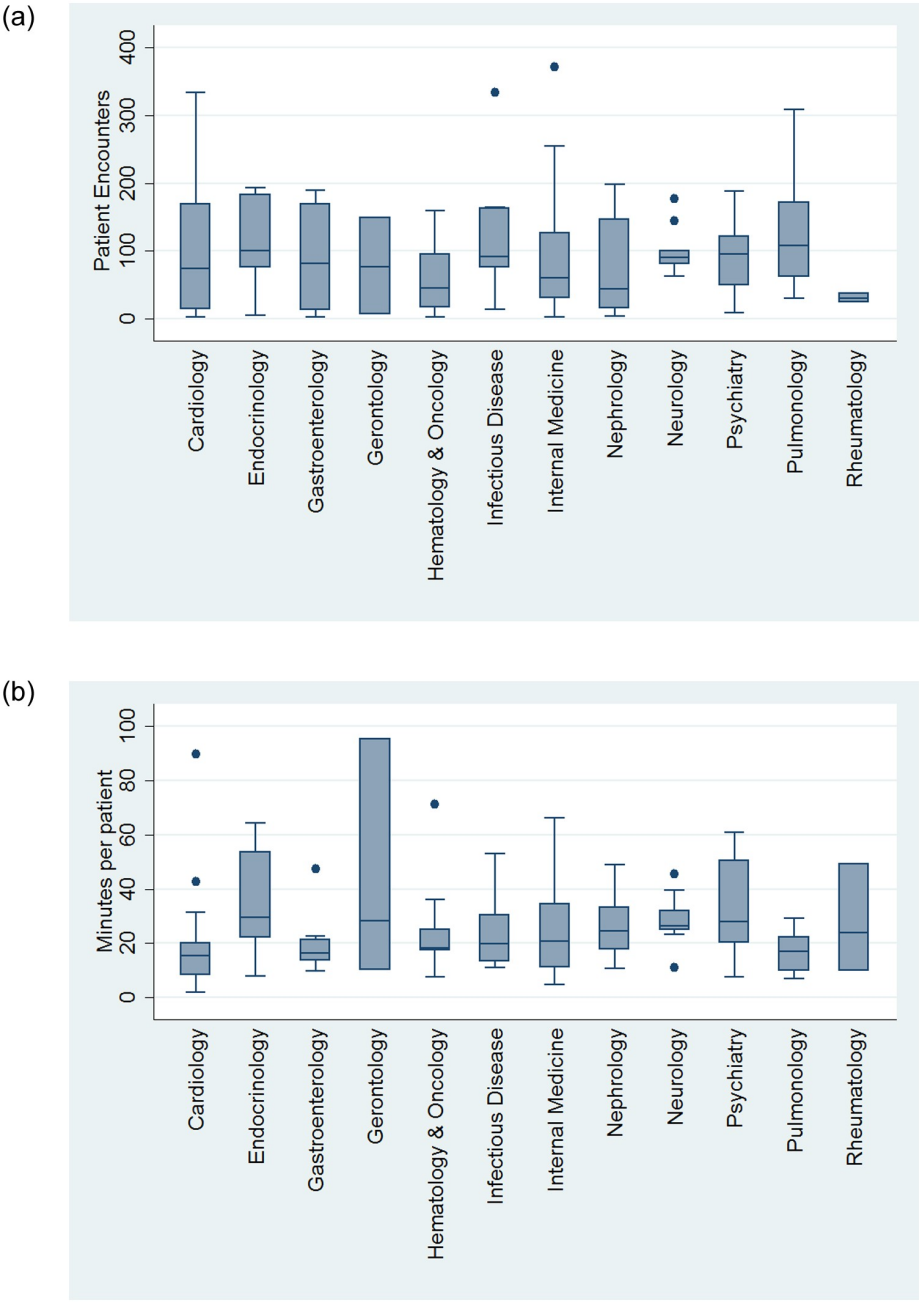
(a) Total number of patient encounters in 2 week period by different specialty. (b) Time spent per patient by different specialties.

Among the attending included in the sub-study, 108 (57%) were private attendings, with Internal Medicine (n = 56, 30%) being the most represented specialtythe median number of patients seen per attending during the 2-week sample period was 75 (25; 127) with median time spent per patient on EHR being 21 (13; 31) minutes. There was no apparent difference in time spent per patient by faculty versus private attending (19 (14; 27) minutes vs. 21 (12;37) minutes, respectively, p = 0.42). Among medicine sub-specialties the greatest number of patients were seen by Endocrinologists 101 (75; 183) spending 30 (22; 55) minutes on EHR per patient (Fig 5a and 5b). For comparison, during the 2-week interval, residents had averaged 111 (±28) patient encounters, spending a mean of 30 (±10) minutes per patient on EHR. Interns were noted to have more encounters with 123 (±14) patients, while spending less time on EHR per patient at 27 (±6) minutes (both p<0.01).

One hundred ninety two alert and awake *in*-patients were approached to participate in a 5 question survey and 167 (87%) completed it. The mean HCAHPS score was 10.9 (±1.9), with 104 (62%) patients reporting a perfect score. There was no correlation between time spent by the treating resident on EHR and patient’s HCAHPS score (β = -.01, p = 0.47). Based on the patient survey, residents spent 10 (±8) minutes in person with each patient per day. There was a trend towards a direct association between time spent on EHR and time spent with each patient (β = 0.09, p = 0.07). The patients reported a mean satisfaction of 83(±23) with relation to the amount of time spent at the bedside by a resident, when asked on a scale of 0–100, with 100 being the most satisfied. There was a direct association between patient satisfaction and resident time at the bedside reported by patients (beta = 0.75, p>0.01). The results of the Oxford Happiness Survey indicated residents to be “somewhat happy” with a mean score of 3.8 (±0.8), however, we did not find a significant correlation between amount of time spent on EHR and resident’s overall happiness (beta = 0.15, p = 0.13).

## Discussion

Our analysis demonstrated that clinicians spent 3.7 hours per day, or 37% of their work day on EHR. There was a marked reduction in EHR time withboth clinician and resident seniority. Despite this improvement, the total time spent on EHR remained exceedingly high amongst even the most experienced physicians. We did not see a correlation between time spent on EHR by physicians with patient or physician satisfaction.

We found thatresidents spent more time on EHR than in direct patient contact per encounter–approximately 37 min compared with 10 minutes, respectively. This objectively demonstrates a shift away from the bedside and a greater emphasis on EHR, demonstrating the demands of modern medicine on physicians at every level of training. An important observation of our analysis is that EHR proficiency improves with progression of training, both in regards to seniority and familiarity of EHR. This may in part be attributed to suboptimal initial EHR training. EHR training at our institution is composed of a single 2-hour session incorporated as part of the hospital orientation followed by self-learning. Continued regular training in EHRwould help counter deficiencies in initial EHR orientations. Furthermore, updates to dated EHR systems may also be of use. Further research comparing different EHR interfaces, in addition to best practices for EHR training are needed.

Our study is inline with a recent report by Young et al., evaluating multiple family medicine residency programs [16]. This study analysed data from 982 patient visits seen by residents and faculty. Based on the reported data, family physicians spent more than 50% of the visit time working on EHR (18.6 (16.6) minutes on EHR with total visit of 35.8 (16.6) minutes) during an ambulatory visit. Despite focusing on inpatient settings we observed similar findings with physicians spending the largest portion of the day on EHR. Additionally, as well as in our study, there was a reduction in EHR time with an increase in PGY level. This finding points to the presence of the “learning curve” and lay the ground for further studies evaluating different formats of the initial EHR training vs continuous EHR modules throughout the training. Another study by Read- Brown et al. analyzed time requirements of EHR use for ophthalmologists in an outpatient setting [17]. This paper found that, on average, an ophthalmologist spent 3.7 hours a day using EHR. Additionally, there was a significant variation among ophthalmologists regarding time requirements and EHR use patterns. In line with our results, EHR use creates significant time burdens for clinicians and better practice to improve users experience should be designed.

The significance of an increasing shift towards EHR is a growing paradigm that cannot be understated, particularly in the current era of healthcare when there is increasing scrutiny on documentation and a ceiling on the number of hours that can be worked by house staff [13, 14, 18]. These increased demands can lead to EHR fatigue and physician burnout. In a survey of a general internal medicine group, 38% reported feeling burn out with 60% citing high documentation pressure and 50% describing too much EHR time at home [19]. Burnout has been linked to an increased risk of resident’s wellbeing [20]. Residents have a nearly fourfold increase in experiencing a major depressive episode during their training compared to similar aged individuals in the U.S. population and it has been observed that 23% of interns had suicidal thoughts [21, 22]. There has been a greater emphasis on resident wellness and reducing physician burnout amongst teaching programs across the country [18, 23]. Reducing EHR time where residents spend nearly two-fifths of their work day may be one step toward this goal.

One limitation of our study was that we chose to only monitor our hospitalist service for attending work hours. This decision was made as their work hours are the most clearly defined with a schedule of working alternating weeks with 12 hour shifts daily. While we observed data in regards to time spent on EHR by faculty and private attendings, it was more challenging to clearly define their work hours.

In conclusion, we found that physicians spend almost 40% of their work day on EHR with Interns spending the most time. Although there is a significant reduction in time spent on EHR with greater training and exposure to EHR, the overall amount of time spent on EHR remains high. We did not identify any factors linked with either physician’s or patient’s satisfaction.

## Supporting information

S1 Table Patient questionnaire. (DOCX)

S2 Table Oxford Happiness questionnaire. (DOCX)

## Abbreviations

ACA: Affordable Care Act
EHR: Electronic HealthRecord
GME: General Medical Education
IQR: Interquartile range
PGY: Postgraduate Year
SD: Standard Deviation
