# Supplementary material for: Analyses of electronic health records utilization in a large community hospital
Source: PLoS One. 2020 Jul 1;15(7):e0233004. doi: 10.1371/journal.pone.0233004 (PMC7329072; doi:10.1371/journal.pone.0233004)
Supplement: S1 Table — (DOCX) [file pone.0233004.s001.docx]

**S1 Table. Patient Questionnaire**

Patient MRN:

Identifier (Initials, Age, Sex):

Location:

Covering Resident Name:

1. During this hospital stay, how often did doctors treat you with courtesy and respect?

1 Never

2 Sometimes

3 Usually

4 Always

2. During this hospital stay, how often did doctors listen carefully to you?

1 Never

2 Sometimes

3 Usually

4 Always

3. During this hospital stay, how often did doctors explain things in a way you could understand?

1 Never

2 Sometimes

3 Usually

4 Always

4. On average, how many minutes per day in total do you feel your resident doctor spends with you in person?

Time in minutes:

5. On a scale of 1-100 (100 being the most satisfied), how satisfied are you with the amount of time spent with you by your resident doctor?

Satisfaction on a scale of 1-100 (100 being the most satisfied):
