# Supplementary material for: Analyses of electronic health records utilization in a large community hospital
Source: PLoS One. 2020 Jul 1;15(7):e0233004. doi: 10.1371/journal.pone.0233004 (PMC7329072; doi:10.1371/journal.pone.0233004)
Supplement: S2 Table — (DOCX) [file pone.0233004.s002.docx]

**S2 Table. Oxford Happiness Questionnaire.**

Below are a number of statements about happiness. Please indicate how much you agree or disagree with each by entering a number in the blank after each statement, according to the following scale:

1 = strongly disagree

2 = moderately disagree

3 = slightly disagree

4 = slightly agree

5 = moderately agree

6 = strongly agree

Please read the statements carefully, some of the questions are phrased positively and others negatively. Don’t take too long over individual questions; there are no “right” or “wrong” answers (and no trick questions). The first answer that comes into your head is probably the right one for you. If you find some of the questions difficult, please give the answer that is true for you in general or for most of the time.

| Question | Answer |
| --- | --- |
| 1. I don’t feel particularly pleased with the way I am. (R) |  |
| 2. I am intensely interested in other people. |  |
| 3. I feel that life is very rewarding. |  |
| 4. I have very warm feelings towards almost everyone. |  |
| 5. I rarely wake up feeling rested. (R) |  |
| 6. I am not particularly optimistic about the future. (R) |  |
| 7. I find most things amusing. |  |
| 8. I am always committed and involved. |  |
| 9. Life is good. |  |
| 10. I do not think that the world is a good place. (R) |  |
| 11. I laugh a lot. |  |
| 12. I am well satisfied about everything in my life. |  |
| 13. I don’t think I look attractive. (R) |  |
| 14. There is a gap between what I would like to do and what I have done. (R) |  |
| 15. I am very happy. |  |
| 16. I find beauty in some things. |  |
| 17. I always have a cheerful effect on others. |  |
| 18. I can fit in (find time for) everything that I want to. |  |
| 19. I feel that I am not especially control of my life. (R) |  |
| 20. I feel able to take anything on. |  |
| 21. I feel fully mentally alert. |  |
| 22. I often experience joy and elation. |  |
| 23. I don’t find it easy to make decisions. (R) |  |
| 24. I don’t have a particular sense of meaning and purpose in my life. (R) |  |
| 25. I feel I have a great deal of energy. |  |
| 26. I usually have a good influence on events. |  |
| 27. I don’t have fun with other people. (R) |  |
| 28. I don’t feel particularly healthy. (R) |  |
| 29. I don’t have particularly happy memories of the past. (R) |  |
